# Time delays in computational models of neuronal and synaptic dynamics

**DOI:** 10.3389/fncom.2025.1700144

**Published:** 2025-11-10

**Authors:** Mojtaba Madadi Asl

**Affiliations:** 1 School of Biological Sciences, Institute for Research in Fundamental Sciences (IPM), Tehran, Iran; 2 Pasargad Institute for Advanced Innovative Solutions (PIAIS), Tehran, Iran

**Keywords:** dendritic delay, axonal delay, neuronal dynamics, synaptic plasticity, computational modeling

## 1 Introduction

Time delays in signal propagation represent a fundamental aspect of neuronal communication that profoundly influences information processing and learning (Gerstner et al., 1993, 1996, 1997). Synaptic delays, in particular, arise from the physical constraints of axonal conduction and dendritic integration. Dendritic and axonal delays within and between brain areas can vary significantly and are not necessarily identical. For instance, dendritic delays tend to be typically shorter than axonal delays, ranging from sub-millisecond to a few milliseconds (Agmon-Snir and Segev, 1993; Schierwagen and Claus, 2001). Axonal delays, on the other hand, can range from a few milliseconds in thalamo-cortical and cortico-tectal connections (Cleland et al., 1976; Swadlow and Weyand, 1987), to as much as tens of milliseconds in cortico-cortical connections (Swadlow, 1990; Stoelzel et al., 2017).

One key aspect of these delays pertains to the modulation of neuronal dynamics. Delays introduce temporal offsets in signal arrival, affecting synchronization and oscillation patterns within neuronal ensembles, as demonstrated in several influential theoretical and computational studies (Ernst et al., 1995; Crook et al., 1997; Ermentrout and Kopell, 1998; Yeung and Strogatz, 1999; Roxin et al., 2005; DHuys et al., 2008; Gilson et al., 2010; Popovych et al., 2011). In plastic networks, however, delays play a dual role: they modulate the temporal dynamics of neuronal activity while simultaneously shaping the adaptive processes underlying neuroplasticity (Gerstner et al., 1993; Debanne et al., 1998; Senn et al., 2002; Morrison et al., 2008; Madadi Asl et al., 2017, 2018b,c).

In oscillatory networks, short delays may decouple synchronous firing, promoting diverse activity states (Lubenov and Siapas, 2008; Kozloski and Cecchi, 2010; Knoblauch et al., 2012; Babadi and Abbott, 2013), whereas longer delays can enhance inter-regional synchronization, facilitating coherent information processing (Knoblauch and Sommer, 2003, 2004). In this way, dendritic and axonal delays may determine phase relationships between neurons. This temporal structuring is crucial for phenomena like gamma oscillations in cortical networks, where precise timing underlies sensory binding and attention mechanisms (Engel and Singer, 2001; Buzsáki and Wang, 2012; Madadi Asl and Valizadeh, 2025). By incorporating these delays, computational models can replicate a rich dynamical repertoire, such as multistable attractors where networks switch between qualitatively different dynamical states based on initial conditions (Kim et al., 1997; Ernst et al., 1998; Madadi Asl et al., 2018a). Such models underscore how delays act as a regulatory mechanism, enabling flexible network reconfiguration as well as transitions between different states (Khoshkhou and Montakhab, 2019; Madadi Asl and Ramezani Akbarabadi, 2023).

Traditional models sometimes simplify or neglect these delays to reduce computational complexity, but their presence is crucial for capturing realistic brain behaviors. For instance, in networks governed by spike-timing-dependent plasticity (STDP) (Gerstner et al., 1996; Markram et al., 1997b; Bi and Poo, 1998), where the synaptic weights are adjusted based on the precise timing of pre- and postsynaptic spikes, delays can alter the effective time lags between spike pairs. This ultimately leads to emergent connectivity patterns that differ markedly from delay-free scenarios (Lubenov and Siapas, 2008; Kozloski and Cecchi, 2010; Knoblauch et al., 2012; Babadi and Abbott, 2013; Madadi Asl et al., 2017), revealing how delay-induced temporal mismatches contribute to learning, memory, and adaptive responses.

More precisely, in STDP frameworks, the outcome of synaptic modification, i.e., long-term depression (LTD) or long-term potentiation (LTP), hinges on the relative spike timing of neuron pairs (Markram et al., 1997b; Bi and Poo, 1998). However, delays modify this timing at the synaptic site, potentially reversing or amplifying expected changes. Analytical studies of two reciprocally coupled neurons demonstrate that the combination of dendritic and axonal delays can shift spike ordering, leading to unconventional STDP outcomes like mutual potentiation or depression in loops (Senn et al., 2002; Morrison et al., 2008; Babadi and Abbott, 2013). This modulation is particularly evident in plastic networks subjected to pair-based STDP, where delays facilitate the preservation of strong bidirectional connections or loop elimination (Madadi Asl et al., 2017, 2018a). These effects scale up in larger recurrent networks, fostering motifs such as strong bidirectional loops when dendritic delays exceed axonal ones, or loosely connected structures in the reverse scenario (Madadi Asl et al., 2017, 2018a).

In this piece, I discuss a crucial aspect of modeling plastic networks, i.e., the differentiation between dendritic and axonal delays. These delays must be taken into account separately in computational models because they contribute uniquely to signal propagation and plasticity. In fact, their sum modulates neuronal dynamics as the total delay affecting signal transmission from the presynaptic neuron to the postsynaptic cell, while their difference determines the time lag perceived at the synapse, influencing the efficacy of synaptic modifications. Incorporating distinct dendritic and axonal delays into computational models significantly improves simulation methodologies. For instance, by accurately representing these delays, models can better replicate the temporal precision observed in biological neuronal networks, leading to more realistic simulations of learning and memory processes. This approach also enables computational studies to explore how disruptions in delay dynamics—such as those caused by neurological disorders—affect brain structure and function. For example, increased axonal delays in conditions like multiple sclerosis, due to demyelination, leads to a reduction of conduction velocity of signals along the axon (Waxman, 2006). This can disrupt reliable signal transmission, leading to impaired oscillatory responses (Lefebvre et al., 2025). Models that account for these delays can advance our understanding of brain function and refine tools for studying complex neuronal systems.

## 2 Incorporating delays into computational models

### 2.1 Delays change spike timings perceived at the synapse

**Figure 1:**
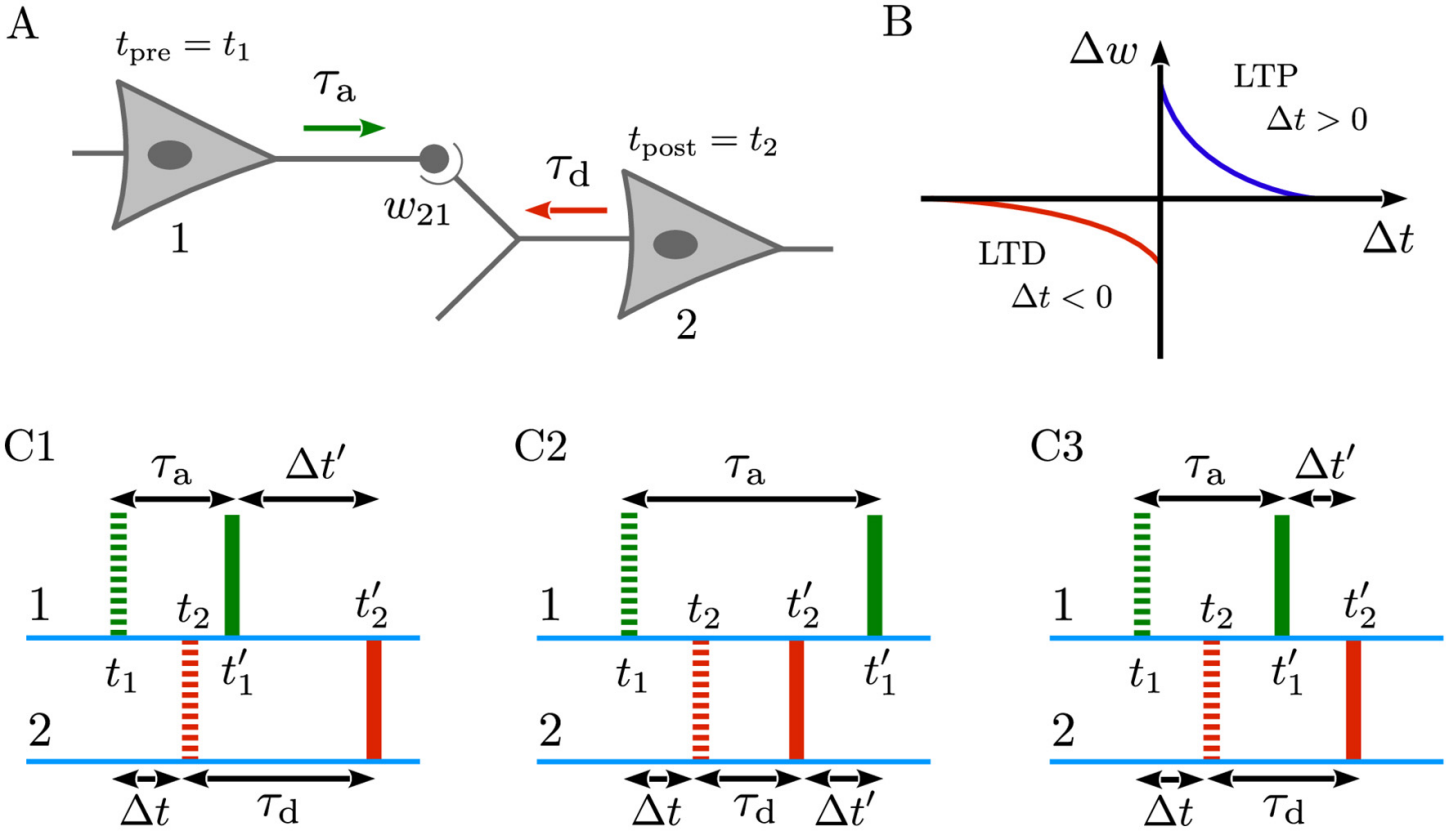
Dendritic and axonal delays change spike timings arriving at the synapse. **(A)** Representation of a neuron pair comprising a presynaptic neuron (1) connected to a postsynaptic neuron (2) with the coupling weight *w*_21_. The presynaptic neuron fires at *t*_pre_ = *t*_1_, and the postsynaptic neuron fires at *t*_post_ = *t*_2_. These spike times arrive at the synapse at $t_{1}^{\prime }=t_{1}+\tau_{\text{a}}$, after axonal delay (τ_a_) in the forward direction (green arrow), and at $t_{2}^{\prime }=t_{2}+\tau_{\text{d}}$, after dendritic delay (τ_d_) in the backward direction (red arrow). **(B)** Schematics of a generic, temporally asymmetric STDP learning window where Δ*t* = *t*_post_−*t*_pre_ = *t*_2_−*t*_1_ is the non-delayed time lag between the pre- and postsynaptic spike pairs, and Δ*w* is the resultant synaptic change. According to Equation 1, pre-before-post spike pairing (Δ*t*>0) induces LTP (Δ*w*>0; blue), whereas post-before-pre pairing (Δ*t* < 0) leads to LTD (Δ*w* < 0; red). **(C1–C3)** Green and red dashed (solid) markers indicate the non-delayed, *t*_1_ and *t*_2_ (delayed, $t_{1}^{\prime }$ and $t_{2}^{\prime }$) forward spike time of the presynaptic neuron and the time of backpropagated potential of the postsynaptic neuron at the synaptic site, respectively. The spike times of the pre- and postsynaptic neurons are separated by a small, non-delayed time lag Δ*t* such that pre-before-post ordering of spikes leads to the potentiation of synapse. Taking into account the effect of dendritic and axonal delays, the delayed time lag perceived at the synapse is Δ*t*′ = Δ*t*+ξ, where ξ = τ_d_−τ_a_. **(C1)** When τ_d_>τ_a_, the pre-before-post orderings of delayed and non-delayed spikes are identical and, therefore, the synapse is potentiated. **(C2)** When τ_d_ < τ_a_, the backpropagated potential of the postsynaptic neuron (solid red) arrives sooner than the forward spike time of the presynaptic neuron (solid green) at the synapse. Thus, according to the STDP rule, the synapse undergoes depression. **(C3)** If τ_d_ = τ_a_, the situation resembles the non-delayed scenario since ξ = 0, and Δ*t*′ = Δ*t*, and the synapse is potentiated due to the pre-before-post spike timing.

As illustrated in Figure 1A, consider that the presynaptic neuron (1) is connected to the postsynaptic neuron (2), characterized by the coupling weight *w*_21_. If the presynaptic neuron fires at *t*_pre_ = *t*_1_ and the postsynaptic neuron fires at *t*_post_ = *t*_2_, both the forward signal propagated along the presynaptic axon (denoted by the green arrow) and the backward signal backpropagated through the dendrite of the postsynaptic neuron (denoted by the red arrow) are essential for inducing synaptic modifications in the form of LTD or LTP (also see Figure 1B) (Sjöström and Häusser, 2006; Clopath and Gerstner, 2010; Froemke et al., 2010). However, when delays are taken into account, the spike times arriving at the synapse (Figures 1C1–C3, solid markers) may significantly differ from those initiated at the cell bodies (Figures 1C1–C3, dashed markers), depending on the trade-off between dendritic and axonal delays. Specifically, as shown in Figures 1C1–C3, when a presynaptic spike is generated at *t*_1_, it must first travel down the axon before reaching the synapse, thus arriving at $t_{1}^{\prime }=t_{1}+\tau_{\text{a}}$, where τ_a_ is the axonal delay. By the same token, a postsynaptic spike generated at *t*_2_ must backpropagate through the dendrite before arriving at the synapse at $t_{2}^{\prime }=t_{2}+\tau_{\text{d}}$, where τ_d_ is the dendritic delay. Consequently, this delay-induced time shift in spikes arrival may significantly affect the outcome of timing-dependent learning paradigms such as STDP (Gerstner et al., 1993; Senn et al., 2002; Morrison et al., 2008; Madadi Asl et al., 2017), as discussed below.

### 2.2 Delay-induced wiring patterns in plastic networks

In plastic networks, the synaptic weights (*w*) between neurons are continually modified based on neuronal activity. Consequently, in the presence of STDP, the synaptic weight between the pre- and postsynaptic neurons in Figure 1A is updated at each step of the simulation (*w*→*w*+Δ*w*) according to the following temporally asymmetric learning window (Bi and Poo, 1998), as depicted in Figure 1B:


$$\text{Δ}w=\{\begin{array}{cc} A_{+}\exp^{-\frac{\left|\text{Δ}t\right|}{\tau_{+}}}, & \text{ Δ}t\geq 0\\ -A_{-}\exp^{-\frac{\left|\text{Δ}t\right|}{\tau_{-}}}, & \text{ Δ}t<0 \end{array}$$ (1)

where Δ*w* is the synaptic change, and Δ*t* = *t*_post_−*t*_pre_ = *t*_2_−*t*_1_ represents the non-delayed time lag between the spikes of the presynaptic neuron (1) and the postsynaptic neuron (2). The parameters *A*_+_(*A*_−_) and τ_+_(τ_−_) denote the learning rate and the effective time window for synaptic potentiation (depression), respectively. The generic form of the STDP learning window is characterized by a greater potentiation amplitude (*A*_+_>*A*_−_) and longer depression time constant (τ_+_ < τ_−_) such that *A*_+_τ_+_<*A*_−_τ_−_ (Bi and Poo, 1998). For biological reasons, synaptic weights in computer simulations are typically constrained within the range *w*_min_<*w*<*w*_max_ by imposing soft or hard bound saturation constraints to ensure they remain within their allowed limits (Gerstner et al., 1996; Kempter et al., 1999; Song et al., 2000; Van Rossum et al., 2000; Rubin et al., 2001; Gütig et al., 2003). According to the STDP rule in Equation 1, when the presynaptic spikes precedes the postsynaptic spike the synapses is strengthened (Δ*t*>0 → Δ*w*>0; blue), whereas the synapse is weakened in the reverse scenario (Δ*t* < 0 → Δ*w* < 0; red) (Markram et al., 1997b). In the absence of delays, when the spike times of the pres- and postsynaptic neurons are separated by a small, non-delayed time lag (as shown by the dashed markers in Figures 1C1–C3), the pre-before-post ordering of spikes leads to the potentiation of synapse.

When dendritic and axonal delays come to play, the perceived time lag at the synapse (Δ*t*′) is not typically the same as the non-delayed time lag (Δ*t*). This difference can be formulated as follows:


$$\begin{array}{l} \begin{array}{c} \text{Δ}t^{\prime }=t_{2}^{\prime }-t_{1}^{\prime }=\left(t_{2}+\tau_{\text{d}}\right)-\left(t_{1}+\tau_{\text{a}}\right),\\ =\left(t_{2}-t_{1}\right)+\left(\tau_{\text{d}}-\tau_{\text{a}}\right)=\text{Δ}t+\xi , \end{array} \end{array}$$ (2)

where ξ = τ_d_−τ_a_, is the difference between dendritic and axonal delays (Madadi Asl et al., 2017), which alters the time lag used by the STDP rule, directly impacting synaptic weight evolution. Therefore, generally, Δ*t* in Equation 1 can be replaced by Δ*t*′. In the absence of delays, ξ = 0 and Δ*t* is recovered. It is important to note that this differs from the sum of dendritic and axonal delays (τ = τ_d_+τ_a_), which indicates the total delay needed for signals to be transmitted from presynaptic neurons to postsynaptic cells. Theoretically, in the presence of delays three different scenarios can occur:

- τ_d_>τ_a_: As illustrated in Figure 1C1, the pre-before-post orderings of delayed and non-delayed spikes are identical and, therefore, the synapse is potentiated but with a different magnitude compared to the non-delayed case, since |Δ*t*′|>|Δ*t*|.
- τ_d_ < τ_a_: In this case, the backpropagated potential of the postsynaptic neuron (Figure 1C2, solid red) arrives sooner than the spike time of the presynaptic neuron (Figure 1C2, solid green) at the synapse. This leads to the depression of the synapse based on the STDP rule in Equation 1.
- τ_d_ = τ_a_: If dendritic and axonal delays are identical in a special case, as shown in Figure 1C3, the delayed and non-delayed time lags are also identical (Δ*t*′ = Δ*t*), and the synapse is potentiated due to the pre-before-post spike timing.

These arguments demonstrate that a synapse shared between two neurons (as in Figure 1A) can be strengthened or weakened, depending on the values of dendritic and axonal delays. Intriguingly, in situations where the two neurons are reciprocally connected to each other even more complex connectivity scenarios can occur, including the emergence of strong bidirectional loops (where both the outgoing and incoming synapses are potentiated), loosely connected structures (where both synapses are depressed), and unidirectional connections (where one synapses is potentiated and the other is depressed) (Madadi Asl et al., 2017). STDP is a local learning rule in which synaptic modifications depend solely on the joint pre- and postsynaptic activity of neurons (Gerstner et al., 1996; Markram et al., 1997b; Bi and Poo, 1998). However, these two-neuron connectivity patterns can build up and significantly influence the global dynamics of large networks of neurons, leading to the emergence of qualitatively different wiring patterns, as shown previousely (Izhikevich et al., 2004; Morrison et al., 2007; Gilson et al., 2009, 2010).

### 2.3 Delays shape the coevolution of neuronal and synaptic dynamics

In computational models of plastic neuronal networks, at least two sets of equations are present: one set describes neuronal dynamics, as in Equation 3a, such as the evolution of the membrane potential of a neuron (*v*) or, more generally, the phase dynamics of neuronal oscillators, while the other describes synaptic dynamics, as in Equation 3b, specifically the time evolution of synaptic weights (*w*). A crucial point is, then, the need to differentiate between dendritic and axonal delays, treating them as distinct components of synaptic delay rather than combining them into a single parameter. First, their sum represents the total delay (τ = τ_d_+τ_a_) required for signal transmission between neurons, which modifies neuronal dynamics in Equation 3a. Second, as argued above, their difference (ξ = τ_d_−τ_a_) represents the delayed spike times of neurons arriving at the synapse which modifies the synaptic dynamics in Equation 3a, determining the emergent connectivity patterns due to plasticity. This leads to the coevolution of the neuronal and synaptic dynamics (Aoki and Aoyagi, 2009; Madadi Asl et al., 2018c), which can be representatively shown as follows:


$$\begin{array}{l} \frac{dv}{dt}=f\left(w,\tau_{d}+\tau_{a}\right), \end{array}$$ (3a)

$$\begin{array}{l} \frac{dw}{dt}=g\left(\text{Δ}t,\tau_{d}-\tau_{a}\right), \end{array}$$ (3b)

where I have deliberately omitted other functionals to emphasize the crucial role of dendritic and axonal delays. Given the initial conditions including the synaptic weights and delays, Equation 3a determines the time course of neuronal activity, as reflected in their spike trains. These spike times are then input into Equation 3b, which calculates the activity-dependent changes in the synaptic weights. These updated synaptic weights are subsequently fed back into Equation 3a to refine the activity dynamics, creating a continuous feedback loop simultaneously shaping neuronal and synaptic dynamics.

## 3 Discussion

A critical emphasis in modeling plastic networks is the necessity to discriminate between dendritic and axonal delays, incorporating them separately rather than as a lumped parameter. In this piece, I clarified the dual purpose of dendritic and axonal delays in computational modeling of neuroplasticity. The first facet of this dual role pertains to the modulation of neuronal dynamics. The sum of dendritic and axonal delays represents the total delay (denoted as τ = τ_d_+τ_a_) required for the transmission of signals from presynaptic neurons to the postsynaptic cells. This parameter modifies neuronal dynamics by introducing temporal offsets in the arrival of the synaptic currents. Transitioning to the synaptic domain, delays exert a profound influence on plasticity mechanisms, forming the second pillar of their dual role. By changing the spike timings arriving at the synapse, dendritic and axonal delays can shape different connectivity patterns in plastic networks. The difference between dendritic and axonal delays (denoted as ξ = τ_d_−τ_a_) plays a key role in this process. This dual functionality not only refines our simulations but also deepens insights into brain processes which crucially depend on temporal resolutions.

Beyond basic dynamics, the inclusion of discriminated delays enhances our understanding of brain function by bridging gaps between theoretical models and empirical observations. Failing to distinguish these can lead to inaccurate predictions; for instance, classic STDP models without delays might overlook the emergence of bidirectional motifs prevalent in cortical networks (Abbott and Nelson, 2000; Song and Abbott, 2001; Lubenov and Siapas, 2008; Kozloski and Cecchi, 2010; Knoblauch et al., 2012; Babadi and Abbott, 2013), leading to discrepancies with experimental data (Markram et al., 1997a; Song et al., 2005). By integrating delays, it is possible to uncover how temporal propagation shapes attractor landscapes - stable states that networks converge to - determining basins of attraction influenced by delay configurations (Ernst et al., 1995; Crook et al., 1997; Ermentrout and Kopell, 1998; Yeung and Strogatz, 1999; Roxin et al., 2005; DHuys et al., 2008). In computational setups, separate parameterization allows for exploring how imbalances, such as prolonged axonal delays in demyelinating diseases, affect plasticity, revealing vulnerabilities in network stability (Lefebvre et al., 2025).

On the methodological front, incorporating dendritic and axonal delays crucially improves simulation methodologies in computational neuroscience. Traditional approaches, often reliant on instantaneous transmission, yield oversimplified dynamics that diverge from biological realism. However, biologically plausible models can benefit from delay inclusion by generating emergent patterns like frustrated antiphase states or preserved recurrent loops (Esfahani et al., 2016; Madadi Asl et al., 2017). Moreover, parameterizing dendritic and axonal delays separately allows for sensitivity analyses, identifying critical thresholds where small changes in ξ precipitate qualitative shifts in network behavior, akin to delay-induced bifurcations in dynamical systems.

This insight extends to computational models with precise time delay representations, providing a framework for hypothesizing therapeutic interventions, such as targeted stimulation to restore optimal timing and shift network dynamics from diseased states to healthy attractors. These advancements are applicable, for instance, to theory-based brain stimulation paradigms where the time-delayed onset of stimuli enables stimulation of two or multiple neuronal ensembles, allowing for precise targeting of specific subsets of neurons with a time delay (Tass, 2003; Tass and Majtanik, 2006; Schmalz and Kumar, 2019; Madadi Asl et al., 2023). In fact, as shown computationally, adjusting stimulation timing to compensate for abnormal neuronal synchronization can desynchronize aberrant oscillations and rewire pathological connectivity patterns, for example, in Parkinson's disease models (Ebert et al., 2014; Manos et al., 2021; Asadi et al., 2024; Madadi Asl and Lea-Carnall, 2025), potentially integrating both short- and long-term synaptic plasticity dynamics during and after electrical stimulation (Madadi Asl and Lankarany, 2025).

Ultimately, multiple plasticity mechanisms at different levels may regulate neuronal and synaptic dynamics in the brain (Zenke et al., 2015). While STDP, a form of Hebbian learning that emphasizes the precise timing of neuronal spikes, is significant, it is crucial to consider other plasticity models influenced by temporal delays. For example, structural plasticity does not strictly conform to Hebbian or anti-Hebbian paradigms (Butz et al., 2009). However, certain forms of structural plasticity may be phenomenologically linked to Hebbian plasticity due to their activity dependence (Fauth and Tetzlaff, 2016). This relationship implies a close interplay between STDP and structural plasticity, where temporal delays in neuronal signaling can significantly influence these processes, potentially altering the timing and effectiveness of synaptic modifications. Delays might affect the synchronization of neuronal firing, thereby impacting the conditions under which structural plasticity is activated, as demonstrated by computational studies employing integrated models of structural plasticity and STDP (Deger et al., 2012, 2018). At another level, synaptic rewiring induced by STDP necessitates stabilization through compensatory mechanisms, such as activity-dependent homeostatic plasticity (Turrigiano and Nelson, 2004; Turrigiano, 2008, 2012). The dynamic adaptation of network activity patterns is closely linked to the STDP rule (Izhikevich and Desai, 2003), as homeostatic plasticity can modulate synaptic strengths in response to changes in overall neuronal activity. Importantly, temporal delays can crucially influence the effectiveness of homeostatic adjustments, as they may disrupt the timely feedback necessary for these mechanisms to respond appropriately, potentially leading to imbalances in synaptic strengths and affecting network stability. Furthermore, learning models such as reinforcement learning with modulated STDP (Florian, 2007; Farries and Fairhall, 2007), which focus on the role of reward signals in shaping behavior and synaptic strengths, operate under different principles and can be affected by delays in feedback. Additionally, rate-based learning models, which depend on the average firing rates of neurons rather than precise spike timing, may also exhibit sensitivity to temporal dynamics (Burkitt et al., 2004; Gilson et al., 2011). Exploring these diverse learning models in the context of delays could provide valuable insights into the broader mechanisms of synaptic plasticity and learning in neuronal networks, highlighting how different strategies adapt to the complexities of temporal interactions.

In conclusion, the dual role of delays in neuronal and synaptic dynamics underscores their critical position in computational models of neuroplasticity. By simultaneously modulating neuronal spike timings perceived at the synaptic site and the strength of synapses, dendritic and axonal delays orchestrate the brain's symphony of activity, while their discrimination ensures models reflect biological intricacies. This approach not only enriches our comprehension of brain function, but also propels simulation methodologies toward greater accuracy and applicability. Discriminating dendritic and axonal delays in computational models will unlock deeper insights into the neuronal code, paving the way for a more realistic and biologically plausible modeling of neuronal and synaptic dynamics.
